# Gondwanan conifer clones imperilled by bushfire

**DOI:** 10.1038/srep33930

**Published:** 2016-09-26

**Authors:** James R. P. Worth, Shota Sakaguchi, Karl D. Rann, Clarence J. W. Bowman, Motomi Ito, Gregory J. Jordan, David M. J. S. Bowman

**Affiliations:** 1 Department of Forest Molecular Genetics and Biotechnology, Forestry and Forest Products Research Institute, 1 Matsunosato, Ibaraki 305–8687, Japan; 2 Graduate School of Human and Environmental Studies, Kyoto University, Yoshida-Nihonmatsu-cho, Sakyo-ku, Kyoto 606-8501, Japan; 3 School of Biological Sciences, University of Tasmania, Private Bag 55, Hobart, Tas. 7001, Australia; 4 Laboratory of Plant Evolution and Biodiversity, Department of General Systems Studies, Graduate School of Arts and Sciences, The University of Tokyo, Tokyo, Japan

## Abstract

Global increases in fire frequency driven by anthropogenic greenhouse emissions and land use change could threaten unique and ancient species by creeping into long-term fire refugia. The perhumid and mountainous western half of Tasmania is a globally important refugium for palaeo-endemic, fire intolerant lineages, especially conifers. Reproductive strategy will be crucial to the resilience of these organisms under warmer, dryer and more fire prone climates. This study analysed clonal versus sexual reproduction in old growth plots dominated by the palaeo-endemic conifer *Athrotaxis cupressoides* (Cupressaceae), a species that lacks any traits to tolerate frequent landscape fire. Across most of the seven plots the amount of sexually derived individuals was lower than clonally derived with, on average, 60% of all stems belonging to the same multi-locus lineage (MLL) (i.e. were clonal). Some MLLs were large spanning over 10 s of metres and consisted of up to 62 stems. The high mortality after fire and the rarity of sexual regeneration means that the range of this fire-intolerant species is likely to contract under enhanced fire regimes and has a limited capacity to disperse via seed to available fire refugia in the landscape.

Forest ecosystems around the world are under severe threat due to climate change and modified land management. In many landscapes increasing occurrence of drought or warming is resulting in synergistic demographic process called ‘interval squeeze’^1^ where fire becomes more frequent while concurrently tree growth and survival rates are reduced and fecundity and seedling establishment decline^2^. Climate change is likely to expose even those forested regions not previously considered vulnerable to the effects of drought and fire^3^. Some at risk areas are restricted mesic and drought free ‘fire refugia’ that harbour suites of fire intolerant plant species, threatening ancient, often species-poor lineages of outstanding biogeographic value with severe range contraction or even extinction.

A prime example of this is the western half of Tasmania, most of which is conserved in a World Heritage Area established in part because of outstanding natural values of the endemic flora that provide a living link to Gondwana. This flora persists in a ‘fire refugium’ that is a global centre of fire sensitive palaeo-endemic communities dominated by conifers that have persisted in the region for tens of millions of years^4^. Encroachment of fire driven by changing climate threatens the ongoing role of this region as a refugium^5^.

This attrition of the Tasmanian palaeo-endemic flora is exemplified by the loss of over 30% of the pre-European distribution of the endemic Cupressaceae conifer *Athrotaxis cupressoides*^6^. Anthropogenic climate change is a major threat to this species, with the loss of tens of hectares of *A. cupressoides* forest from unprecedented fire events in 2016 in which 80 dry lightning ignited fires occurred across western Tasmania following some of the driest and warmest spring and summer months on record^5^. This study investigates the reproductive strategies of *Athrotaxis cupressoides* to illuminate why this species has such limited recovery following wildfire and how, to date, it has been able to dominate some important fire refugia in Tasmania.

*Athrotaxis cupressoides* is one of only two species in the ancient (~150 million year old) genus *Athrotaxis*^7^,^8^. The species is very long lived – with individual stems reaching ages of over 1000 years – and slow growing. For example, at Mt Field individuals one metre in height are on average 55 years old^9^. The species is currently restricted to interglacial refugia in fire protected, high rainfall regions in the mountains and plateaus of western, southern and central Tasmania. This species is unusual for a conifer in its capacity to propagate by root suckering^10^,^11^. Thus, reproduction in stands of this species may be dominated by clonal growth whereby the parental genotype (genet) expands via production of vegetative modules (ramets).

Both *A. cupressoides* adults and juveniles are killed by all but the lowest intensity fires, reflecting the absence of any specialised fire tolerating traits, such as aerial or soil seedbanks, epicormic resprouting or fire resistanct bark. Intense grazing by mammals of any regeneration from either seeds, that can disperse a maximum of two tree heights from the parent tree^12^, or root suckers from any surviving adult trees reduces the likelihood of stand recovering following fire^13^,^10^. Regeneration failure is amplified given that the species only episodically produces seed crops (i.e. masting)^10^ and that the risk of fire increases in burnt stands because of a switch to more flammable understoreys^13^. These factors explain why the species is restricted to fire proof landscape settings within the widespread matrix of flammable vegetation dominated by *Eucalyptus*^14^ and is unable to re-establish following landscape fires. Indeed, in the last 200 years large tracts of conifer rich palaeoendemic vegetation have been lost after European induced fire events that infiltrated areas that were not burnt under Aboriginal fire management^13^,^15^.

We use microsatellite markers^16^ to investigate the importance of clonality in the persistence of the species in seven stands across the species’ range (Table 1). We aim to (1) test whether the species can form extensive clones; (2) to assess the frequency of sexual reproduction of natural stands across a range of different environments; and (3) to estimate the level of clonal vs sexual reproduction between tall stems (over 1.5 m) and short stems (under 1.5 m).

## Results

### Clonal diversity

The importance of clonality varied between all seven plots as demonstrated by the average genotypic richness (*R*) (average = 0.52) varying from 0.99 at Pine Lake (that is, 99% of individuals belonged to different Multi-Locus Lineages (MLLs)) to the lowest value, which was observed at the Tyndall Range (*R* = 0.16) (Table S1). Three plots (Cradle Mountain (*R* = 0.33), Forgotten Lake (*R* = 0.39) and Tarn Shelf (*R* = 0.37)) were below the overall average while Lake McKenzie (*R* = 0.56) and The Labyrinth (*R* = 0.82) were above average (Table S1). For further detail of the plot based clonal diversity results see Table S1 of the Supplementary Materials.

**Figure 1:**
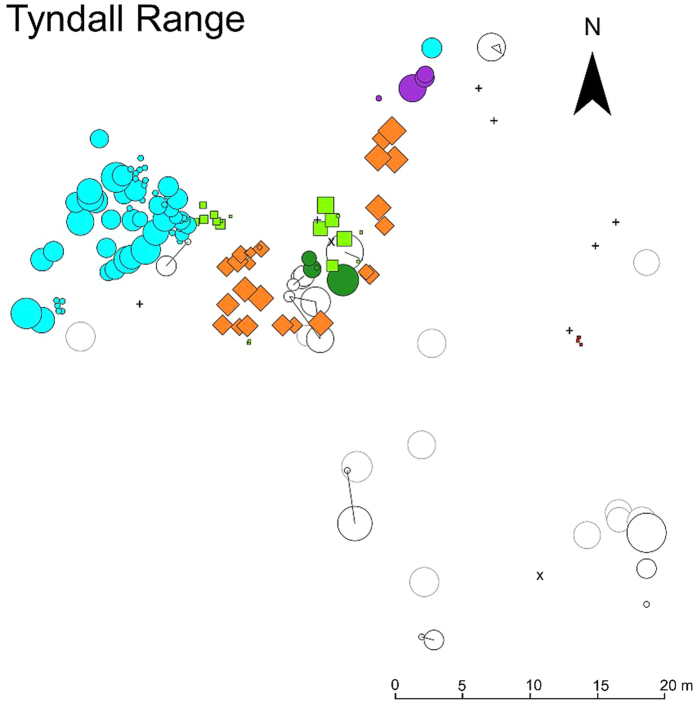
The spatial distribution of the ramets of each identified multi-locus lineages (MLLs) and singletons in the Tyndall Range plot. The different MLLs with over 3 ramets are shown using combinations of different colours and shapes while those with two or three ramets are shown as white black outlined circles connected by lines. Singleton MLLs are shown as white circles with grey outline. Two dead stems are indicated by ‘x’ symbols and seven samples with missing data by crosses. The size of the shapes is proportional to the stem diameter in this plot with the diameter being a maximum 78.7 cm to a minimum of 2 cm, respectively.

The most ramets per a single MLL was found in the Tyndall Range plot consisting of 62 stems with two other MLLs in the Tyndall Range plot consisting of over 19 ramets (Fig. 1). In other plots, large MLLs were found at Cradle Mountain (maximum 20 ramets) and Forgotten Lake (21 ramets) (for clonal maps of all plots other than the Tyndall Range see Supplementary Materials). For MLLs that were found in more than one individual the average number of ramets per MLL was 4.6 varying from 2 at Pine Lake to 10.46 at Tyndall Range (Table 2).

### Spatial structure of clones

The degree to which the clones intermixed with each other varied considerably between plots. Thus, the aggregation index (*Ac*) which measures the degree of aggregation versus intermingling of MLLs, was also highly variable across plots (Fig. 1 and Figures S2–S6). The highest *Ac* values indicating a lack of intermingling of MLLs of over 0.76 were observed at Tarn Shelf and Tyndall Range (Table 2). In contrast, The Labyrinth, Forgotten Lake and Cradle Mountain plots had *Ac* values under 0.3. The maximum distance between ramets of the same MLL (clonal subrange) was remarkably consistent across the plots with the maximum distance between ramets of the same MLL for all plots being over 25 m, apart from Pine Lake where only one multi-ramet MLL was found (Table 2).

**Figure 2:**
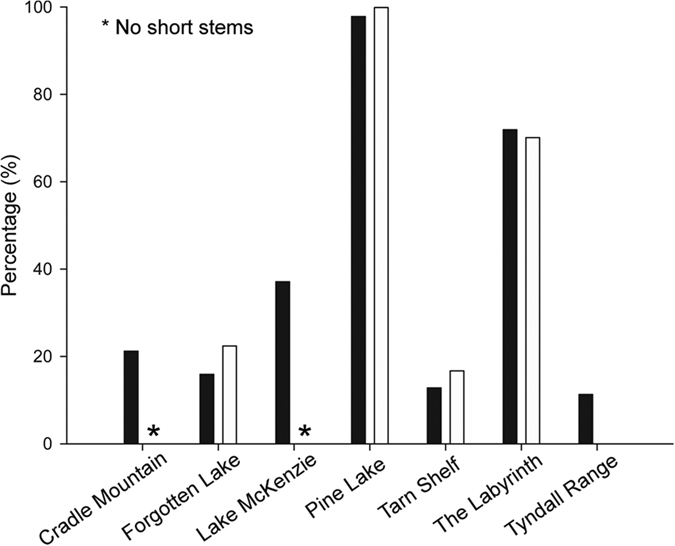
The percentage of tall stems versus short stems belonging to an MLL found in only one individual (i.e. a singleton) at each plot. Black filled bars represent tall stems (over 1.5 m) and white filled bars short stems (under 1.5 m). These values were calculated using a threshold of 0 with all six microsatellite loci. No short stems were observed at Cradle Mountain or Lake McKenzie. No singleton short stems were observed at Tyndall Range.

Apart from The Labyrinth and Pine Lake, all plots were found to have more than 34% of individuals belonging to a multi-ramet MLL (Table 2). This number increased in the tall stem size class (stems over 1.5 m) with all plots, apart from The Labyrinth and Pine Lake, having over 60% of tall stems belonging to a multi-ramet MLL (Table S2). The same pattern was observed in the short stem class (stems under 1.5 m) with over 77% of all short stems at Tyndall Range, Forgotten Lake and Tarn Shelf belonging to a multi-ramet MLL (Table S2 and Fig. 2). In contrast, the Labyrinth had only 29.9% of short stems belonging to multi-ramet MLLs. All multi-ramet MLLs found across the plots were also found in the tall stem class, with the exception of the Tyndall Range (Fig. 1) and The Labyrinth (Figure S6) which may be due to short stems growing from a buried stem/root or possibly unsampled stems.

## Discussion

This study shows that reproduction in *Athrotaxis cupressoides* was dominated by asexual growth, and this process can result in clonal populations extending tens of metres. For five of the seven clonal plots the majority of tall stems and short stems belonged to an existing multi-locus lineage within each plot. The largest clone at Tyndall Range consisted of 62 stems, which is the largest clone reported for a root suckering conifer and rivals the largest clone (in terms of ramet number) known in conifers being an 82 ramet clone observed in the layering conifer *Thujopsis dolobrata* endemic to Japan^17^. Many of the clones of *A. cupressoides* must be of great age although it is difficult to accurately determine the age of each clone^18^ because they may have lost the original ramet that developed from the genet’s seedling establishment. These results suggest that under the current climate seedling establishment is much more infrequent than suggested by the high density of young stems in some populations. Given the likely great age of the largest clones, at least some individual may be survivors from the first seedlings that established on newly exposed substrates following ice-sheet retreat at the end of the LGM.

The fire-protected habitat of *Athrotaxis cupressoides* presents a number of constraints to the dispersal potential of the species to new suitable areas under climate change. *Athrotaxis cupressoides* commonly occurs in fire refugia that are typically poorly drained, and often consist of sphagnum bogs that are an ideal medium for root suckering^10^. For instance, the prolific clonal production observed at Forgotten Lake, with 158 clonal short stems identified within a 20 m radius, occurred in a sphagnum bog. Thus, the species restricted seed dispersal, requirement of high soil moisture for seedling establishment such as peat beds^10^ and prevalence of clonal growth in such sites clearly constrains this species’ ability to locally expand its geographic range. In contrast, clonal reproduction was almost absent at the only plot that did not occur on a poorly drained plot (Pine Lake). However, successful establishment of seedlings across the species range appears to be restricted by browsing pressure of both native and introduced mammals^13^,^10^. These constraints suggest that during warm interglacials populations may persist as clones in scattered refugia and reproduction via seed may be favoured under colder and generally less fire prone glacial climates. An expectation of this hypothesis is that under current climate warming there will be ongoing loss of existing populations reducing the genetic diversity and increasing the risk of extinction of this ancient species. Interventions such as assisted migration to fire-free moist environments both within and outside Tasmania, and sustained fire suppression in its native habitat, will increase the survival prospects of this species and its genetic diversity.

Overall, this study demonstrates the precarious status of a fire intolerant species even in long-term fire refugium. The lack of sexual reproduction coupled with browsing pressure reducing the chance of survival of any seedlings to maturity means that *A. cupressoides* is ill equipped to cope with the likely encroachment of fire due to anthropogenic driven climate change.

## Methods

### The study species

*Athrotaxis cupressoides* ranges from a tall tree up to 30 m to a small dwarfed shrub above the treeline^19^. Large individuals have basal diameters exceeding 1 m^6^. The species is most common in the Central Plateau region of Tasmania where it can form extensive stands. Smaller stands occur scattered in many other high altitude (mostly >900 m above sea level) areas of western and southern Tasmania. Where the species occurs it usually is the dominant tree and appears to undergo continuous gap regeneration^13^ although most stands contain few or no young individuals.

### Sampling

We investigated clonality in the species in seven old growth stands that have never been logged (Table 1). These stands were chosen to represent the range of environmental variation across the species range. Within each stand we positioned a circular plot of 20 m radius in a location typical of the overall stand and sampled nearly all stems of *A. cupressoides*. Circular plots are ideal for estimating clonal diversity because they minimize the perimeter to area ratio, thus reducing the chance of falsely assigning individuals at the perimeter to unique genotypes^20^. Within the centre of each plot, a sighting compass on a tripod was placed to determine the angle and distance to each tree using an ultrasound distance measuring device. In this way, we constructed a detailed map of the distribution of all individual stems (defined as independently emergent from the ground surface). We defined tall stems as those over 1.5 m tall and all other stems under 1.5 m tall were considered to be short stems. Any rare individuals under 15 cm in height were not sampled due to their delicate nature. Stems were measured from the side of the stem closest to the sighting compass, with their location positioned according to their base - therefore the mapping denotes the locations of the bases of the trees not their crowns. The locations of all dead individuals and non-sampled trees were also recorded. For almost all live individuals in each plot a leaf sample was taken for genetic analysis and the basal diameter was recorded which should be a reliable indicator of tree age, as stem diameter is strongly correlated with age in this species^10^.

### Data analyses

We extracted DNA using a DNeasy 96 Plant Kit (Qiagen). Six microsatellite primers designed specifically for *A. cupressoides*^16^ were amplified in all individuals using the method described in Worth *et al*.^8^ and ran on an ABI 3600 Analyzer. The microsatellite loci included the three most variable loci observed in a range-wide study of *A. cupressoides* (Worth *et al. in review*) (Ath_10577 with 21 alleles, Ath_10333 with 23 alleles and a mitochondrial (mtDNA) locus Ath_0007753 with 12 alleles). Allele binning was undertaken using the software GENEMARKER (SoftGenetics, State College, PA) and checked by eye for accuracy.

The program Genodive^21^ was used to evaluate the clonal diversity within each plot. Given that individuals of the same clone may in fact differ due to somatic mutations or genotyping errors a threshold based method was used to determine clonal diversity, with a threshold being the maximum genetic distance that is allowed between two individuals to still be classified as the same clone. A threshold of 0 identifies identical individuals only (i.e. multi-locus genotypes (MLGs)) while thresholds above 0 identify those individuals belonging to the same multi-locus lineage (MLLs). Hereafter MLGs and MLLs are referred to as MLLs for the sake of simplicity. Threshold values from 0 to 5 were used to evaluate the impact that different thresholds had on estimates of clonal diversity for each plot following the recommendations of ref. 21. Clone assignment was undertaken using a stepwise mutation model (SMM). Overall, the six microsatellite loci were reliable in identifying the multi locus genotypes in each plot with, apart from Forgotten Lake, the number of MLGs identified being similar when five or six loci were used (Figure S1). A threshold of 0 resulted in the same number of multi locus lineages (MLLs) estimated as for a threshold of 1 (i.e. one stepwise mutational difference) for all seven plots when all six loci were used (see Table S3). Using a threshold of 2 resulted in no change in MLL numbers for four plots (Cradle Mountain, Tarn Shelf, The Labyrinth and Tyndall Range), while the number of MLLs was reduced from 170 to 165 at Forgotten Lake, from 98 to 96 at Lake McKenzie and from 88 to 87 at Pine Lake, indicating that the estimated number of MLLs is relatively robust from 0 to 2 (Table S3). The probability of observing recurrent MLLs due to sexual reproduction was not calculated given it is known to be inaccurate as its value depends on allele frequencies of the whole population which cannot be known^20^.

Clonal diversity of each plot was assessed using Genodive using the following statistics: the number of MLLs; the effective number of genotypes (eff); Simpson’s diversity index corrected for sample size (div) (i.e. the probability that two individuals drawn at random will belong to the same clone); Shannon index (shc) corrected for sample size^22^ and the modified index of genotypic richness *R* (where *R* = *G* − 1/*N* − 1 with *G* = to the number of MLLs and *N* the number of samples)^23^. The *R* value will always be 0 for a plot consisting of a single MLL and one for maximal genetic diversity (i.e. all individuals are distinct MLLs).

Clonal evenness was assessed using the evenness statistic (eve = eff/num) which is an indicator of how evenly the genotypes are distributed over the population with an evenness value of 1 indicating that all genotypes have equal frequencies.

To determine whether the sampling design suffered from edge effects we tested whether unique or rare MLLs located near the periphery of each plot may derive from edge effects rather than small clonal lineage size using Genclone. The edge effect index shows whether MLLs tend to be distributed towards the edge of the sampling area. A test of edge effect found no evidence that the number of MLLs was higher at the edge of the lots versus the centre (results not shown). To examine the degree of aggregation versus intermingling of MLLs the aggregation index was calculated in Genclone^24^. The index ranges from 0 when the probability of the nearest neighbour does not differ from the overall average to 1 when all nearest neighbours share the same clone. Lastly, clonal subrange, the largest distance between individuals of the same MLL observed in the plots, was also calculated in Genclone.

## Additional Information

**How to cite this article**: Worth, J. R. P. *et al*. Gondwanan conifer clones imperilled by bushfire. *Sci. Rep.* **6**, 33930; doi: 10.1038/srep33930 (2016).

## Supplementary Material

Supplementary Information

## Figures and Tables

**Table 1:** Details of the seven *Athrotaxis cupressoides* stands from which clonal plots were sampled, including altitude, slope, evidence of fire, drainage, the number of samples collected in each size class and the number of samples not included in the analyses due to missing data.

Site	Altitude (m)	Slope (degrees)	Evidence of fire*	Drainage (1 = wet, 0 = dry)	Samples All	Tall stems	Short stems	Not sampled/missing Tall Stems	Not sampled/missing Short stems
Cradle Mountain	946	12	0	1	104	104	0	10	0
Forgotten Lake	977	1	0	1	437	232	205	11	5
Lake McKenzie	1138	4	1	1	175	175	0	20	0
Pine Lake	1206	4	1	0	89	88	1	7	0
Tarn Shelf	1211	11	0	1	71	47	24	2	0
The Labyrinth	1160	7	0	1	99	32	67	0	8
Tyndall Range	1005	0	0	1	147	97	50	6	1
Average	—	—	—	—	160	111	50	8	3

^*^Evidence of fire included fire killed trees and fire scars.

**Table 2:** Clonal size and structure calculated using a genetic distance threshold of 0 including: the number of multi-locus lineages (MLLs) found that had more than one ramet (with the percentage of multi-ramets MLLs compared to the overall number of MLLs identified in brackets); the average number of ramets per multi-ramet MLL; the maximum number of ramets per MLL observed in each plot; the maximum distance between ramets of the same MLL in metres (clonal subrange) and the aggregation index (*Ac*).

Plot	No. multi-MLL	Average no. ramets per multi-ramet MLL	Max. ramets per MLL	Clonal subrange (m)	*Ac*
Cradle Mtn	13 (37%)	6.31	20	32.5	0.25
Forgotten Lake	87 (51%)	4.07	21	38.2	0.30
Lake McKenzie	33 (34%)	3.43	9	26.8	0.43
Tarn Shelf	17 (63%)	3.59	9	27.6	0.79
Pine Lake	1 (1%)	2	2	0.48	0.10
The Labyrinth	11 (13%)	2.64	5	27.2	0.27
Tyndall Range	13 (54%)	10.46	62	28.9	0.77
Average	25	4.64	18.29	25.95	0.43
