# Supplementary material for: Gondwanan conifer clones imperilled by bushfire
Source: Sci Rep. 2016 Sep 26;6:33930. doi: 10.1038/srep33930 (PMC5036195; doi:10.1038/srep33930)
Supplement: Supplementary Information [file srep33930-s1.pdf]

Gondwanan conifer clones imperilled by bushfire

James R. P. Worth[1,*], Shota Sakaguchi[2], Karl D. Rann[3], Clarence J. W. Bowman[3], Motomi Ito[4], Gregory J. Jordan[3], David M. J. S. Bowman[3]

[1]Department of Forest Genetics, Forestry and Forest Products Research Institute, 1 Matsunosato, Ibaraki 305–8687, Japan.

[2] Graduate School of Human and Environmental Studies, Kyoto University, Yoshida-Nihonmatsu-cho, Sakyo-ku, Kyoto 606-8501, Japan.

[3]School of Biological Sciences, University of Tasmania, Private Bag 55, Hobart, Tas. 7001, Australia.

[4]Laboratory of Plant Evolution and Biodiversity, Department of General Systems Studies, Graduate School of Arts and Sciences, The University of Tokyo, Tokyo, Japan

* Correspondence and requests for materials should be addressed to J. R. P. W (email: jrpw2326@affrc.go.jp)

Supplementary Table S1. Clonal diversity and evenness statistics of the seven plots and a threshold for multi-locus lineage (MLL) identification of 0, including the effective number of genotypes (eff), Simpson's diversity index corrected for samples size, Shannon index corrected for sample size (Chao & Shen 2003), the modified index of genotypic richness *R* (Dorken & Eckert 2001) and evenness statistic (eve = eff / num) which is an indicator of how evenly the genotypes are distributed over the population with an evenness value of 1 indicating that all genotypes have equal frequencies.

Population	*N*	No. MLLs	eff	Simpson's diversity index	Shannon index	*R*	eve
Cradle Mountain	104	35	12.52	0.93	1.43	0.33	0.36
Forgotten Lake	437	170	75.93	0.99	2.19	0.39	0.45
Lake McKenzie	175	98	55.99	0.99	2.09	0.56	0.57
Tarn Shelf	71	27	17.44	0.96	1.44	0.37	0.65
Pine Lake	89	88	87.04	1.00	3.59	0.99	0.99
The Labyrinth	99	81	62.43	0.99	2.40	0.82	0.77
Tyndall Range	147	24	4.35	0.78	0.99	0.16	0.18
Average	160	74.7	45.10	0.95	2.02	0.52	0.57

Supplementary Table S2. The percentage of tall stems versus short stems assigned to a multilocus genotype found in more than one individual in the plot or assigned to a MLL only found in one individual (i.e. a singleton). These values were calculated using a threshold of 0 with all six loci. There were no short stems observed at Cradle Mountain and at Lake McKenzie.

Site	% Tall stems member of multi-MLL	% Tall stems singleton	% short stems member of multi-MLL	% Short stems singleton
Cradle Mountain	78.8	21.2	-	-
Forgotten Lake	84.1	15.9	77.6	22.4
Lake McKenzie	62.9	37.1	-	-
Pine Lake	2.2	97.8	0.0	100.0
Tarn Shelf	87.2	12.8	83.3	16.7
The Labyrinth	28.1	71.9	29.9	70.1
Tyndall Range	88.7	11.3	100.0	0.0
Average	61.7	38.3	58.2	41.8

Supplementary Table S3. Clonal diversity values under thresholds 0 to 5 as calculated in Genodrive.

Population	Threshold	No. MLLs	eff	Simpson's diversity index	evenness	Shannons index
Cradle Mountain	0	35	12.519	0.929	0.358	1.43
	1	35	12.519	0.929	0.358	1.43
	2	35	12.519	0.929	0.358	1.43
	3	35	12.519	0.929	0.358	1.43
	4	34	12.461	0.929	0.366	1.413
	5	34	12.461	0.929	0.366	1.413
Forgotten Lake	0	170	75.932	0.989	0.447	2.192
	1	170	75.932	0.989	0.447	2.192
	2	165	73.876	0.989	0.448	2.173
	3	141	48.31	0.982	0.343	2.032
	4	124	28.696	0.967	0.231	1.892
	5	123	28.678	0.967	0.233	1.888
Lake McKenzie	0	98	55.987	0.988	0.571	2.094
	1	98	55.987	0.988	0.571	2.094
	2	96	55.38	0.988	0.577	2.077
	3	95	54.785	0.987	0.577	2.067
	4	95	54.785	0.987	0.577	2.067
	5	92	52.893	0.987	0.575	2.033
Pine Lake	0	88	87.044	1	0.989	3.594
	1	88	87.044	1	0.989	3.594
	2	87	85.172	0.999	0.979	3.289
	3	85	81.66	0.999	0.961	2.982
	4	85	81.66	0.999	0.961	2.982
	5	85	81.66	0.999	0.961	2.982
Tarn Shelf	0	27	17.443	0.956	0.646	1.444
	1	27	17.443	0.956	0.646	1.444
	2	27	17.443	0.956	0.646	1.444
	3	22	11.227	0.924	0.51	1.28
	4	22	11.227	0.924	0.51	1.28
	5	22	11.227	0.924	0.51	1.28
The Labyrinth	0	81	62.427	0.994	0.771	2.398
	1	81	62.427	0.994	0.771	2.398
	2	81	62.427	0.994	0.771	2.398
	3	81	62.427	0.994	0.771	2.398
	4	78	59.4	0.993	0.762	2.313
	5	78	59.4	0.993	0.762	2.313
Tyndall Range	0	24	4.347	0.775	0.181	0.992
	1	24	4.347	0.775	0.181	0.992
	2	24	4.347	0.775	0.181	0.992
	3	24	4.347	0.775	0.181	0.992
	4	22	4.007	0.756	0.182	0.938
	5	21	4.005	0.755	0.191	0.925

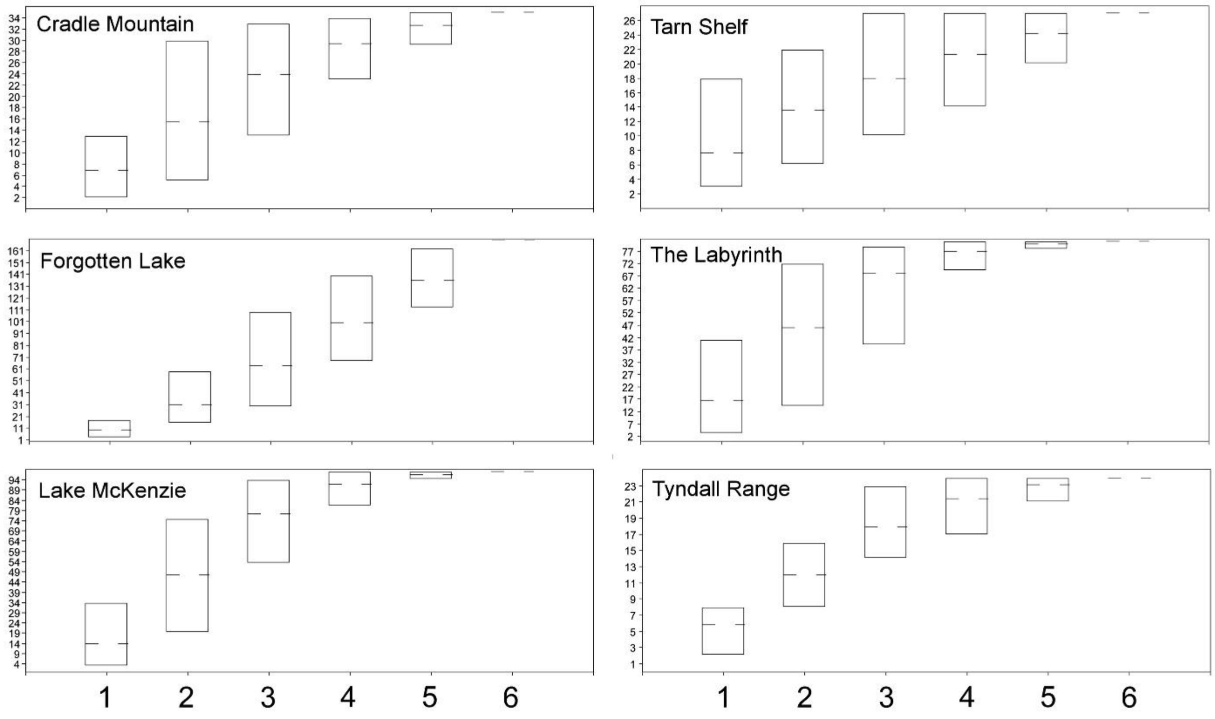

Supplementary Figure S1. The number of multi-locus genotypes (MLGs) identified by sub-sampling of the six microsatellite loci for 6 plots. Pine Lake is not shown due to the near absence of clonality in this plot.

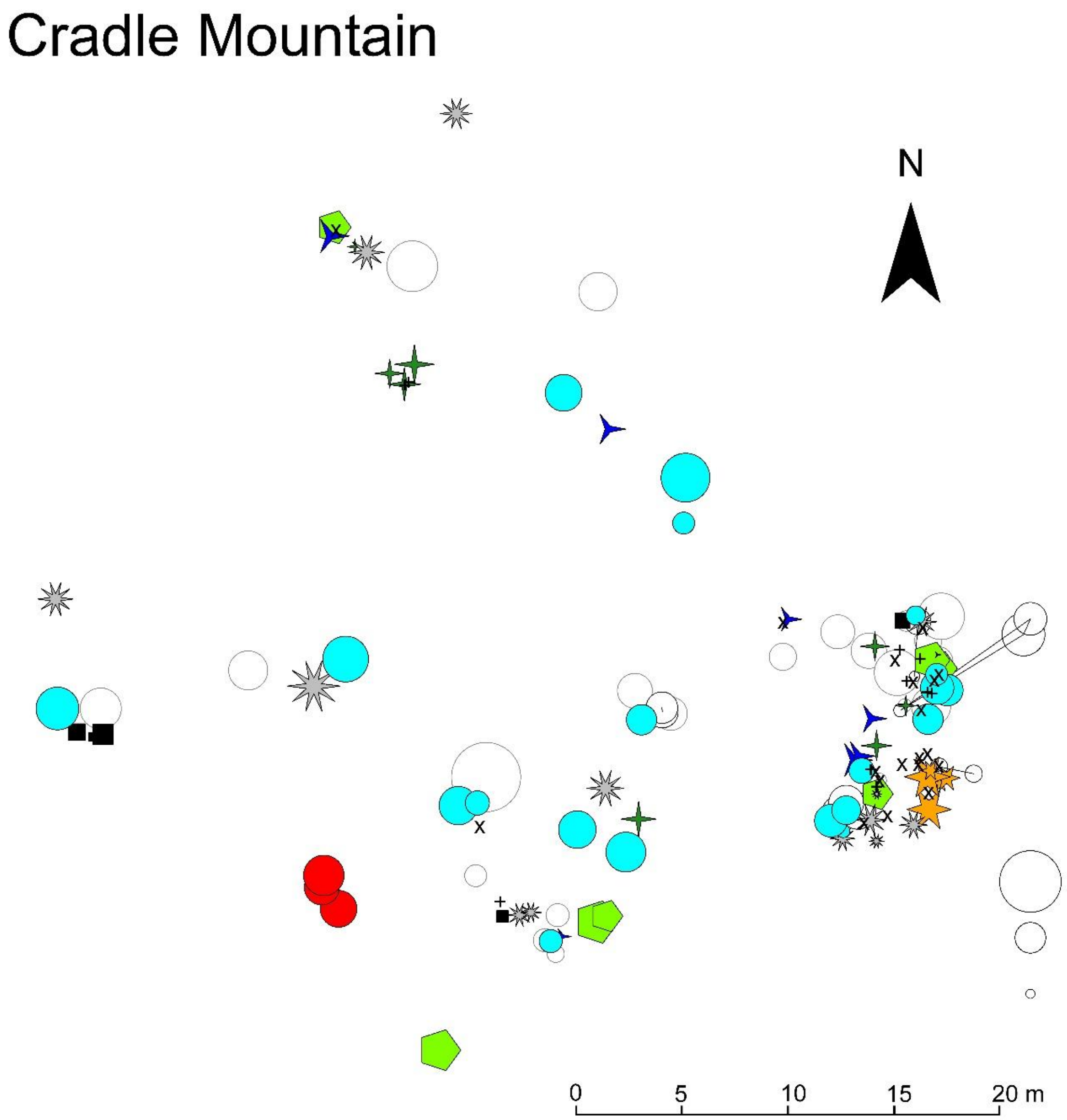

Supplementary Figure S2. The spatial distribution of the ramets of each identified multi-locus lineages (MLLs) and singletons in the Cradle Mountain plot. The different MLLs with over 4 ramets are shown using different colours and shapes while those with under 3 ramets are shown as white black outlined circles connected by lines. Singleton MLLs are shown as white circles with grey outline. Nineteen dead stems are indicated by ‘x’ symbols and ten individuals with missing data are shown by crosses. The size of the shapes is proportional to the stem diameter with the diameter being a maximum 177.2 cm in this plot to a minimum of 2 cm.

.

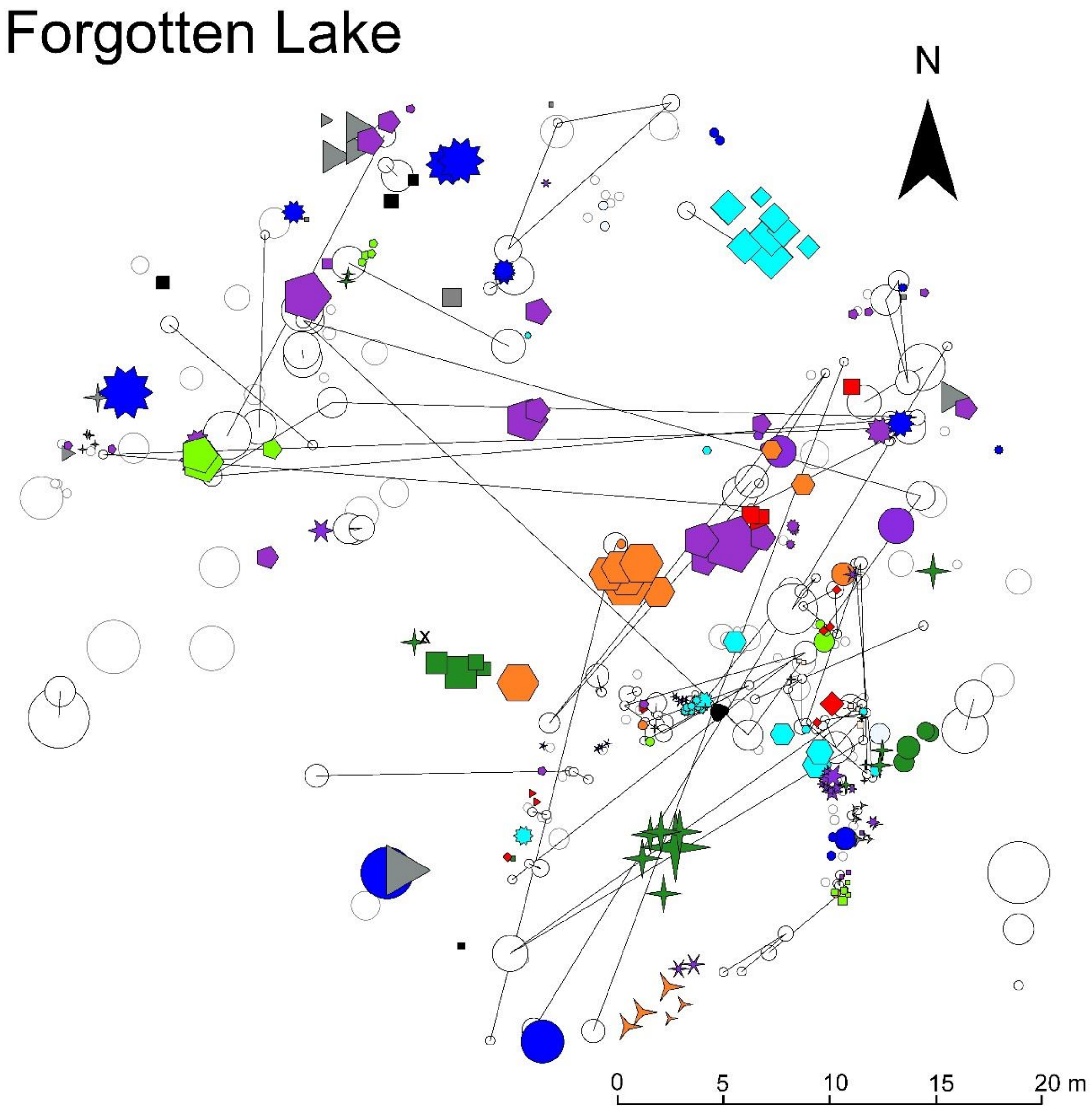

Supplementary Figure S3. The spatial distribution of the ramets of each identified multi-locus lineages (MLLs) and singletons in the Forgotten Lake plot. See caption of Figure S2 for explanation of figure. One dead stems is indicated by an 'x' symbol and 16 individuals with missing data are shown by crosses. The size of the shapes is proportional to the stem diameter with the diameter being a maximum 187.7 cm in this plot to a minimum of 2cm.

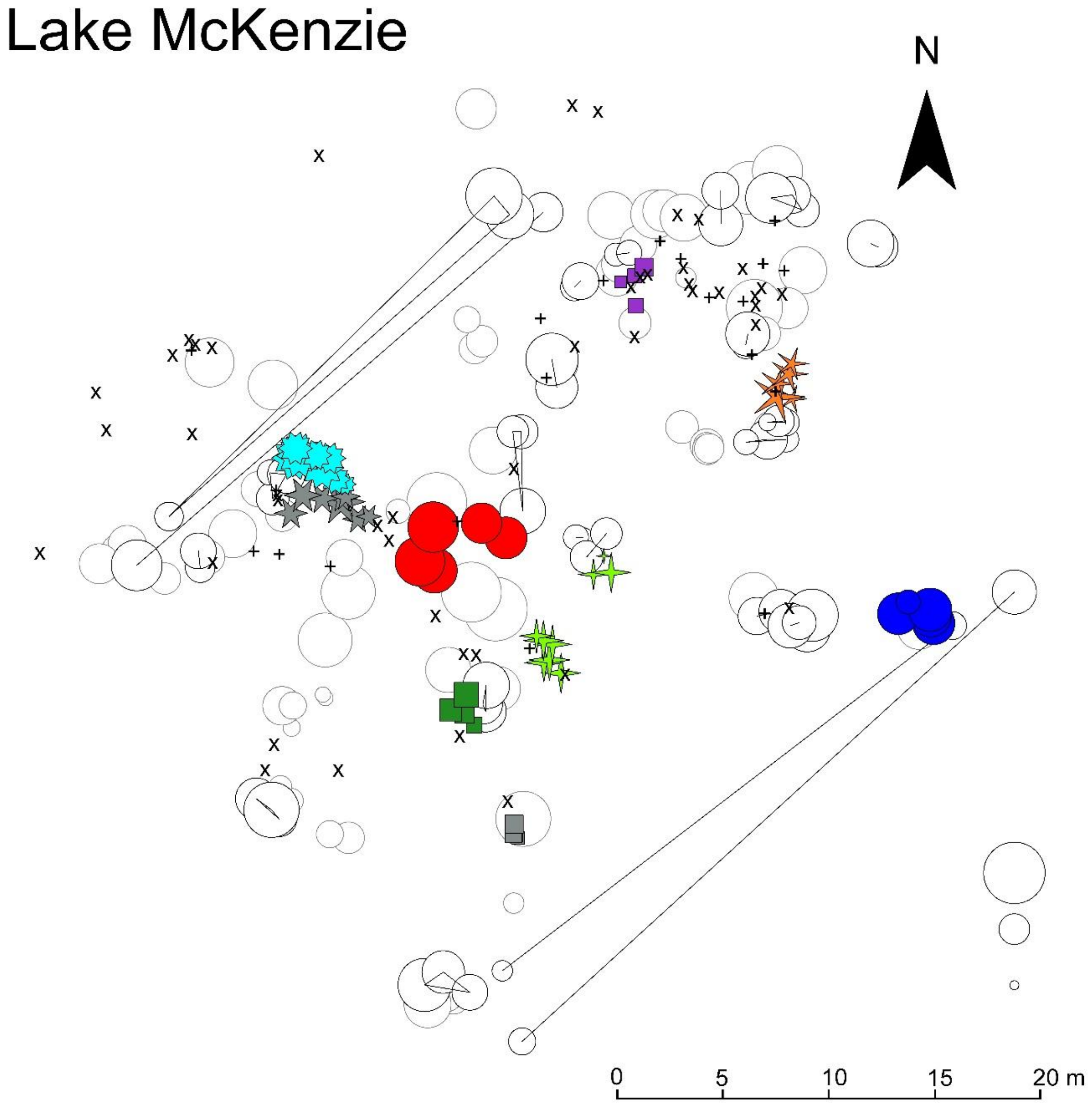

Supplementary Figure S4. The spatial distribution of the ramets of each identified multi-locus lineages (MLLs) and singletons in the Lake McKenzie plot. See caption of Figure S2 for explanation of figure. Forty five dead stems are indicated by 'x' symbols and 20 individuals with missing data are shown by crosses. The size of the shapes is proportional to the stem diameter with the diameter being a maximum 118.7 cm in this plot to a minimum of 2 cm.

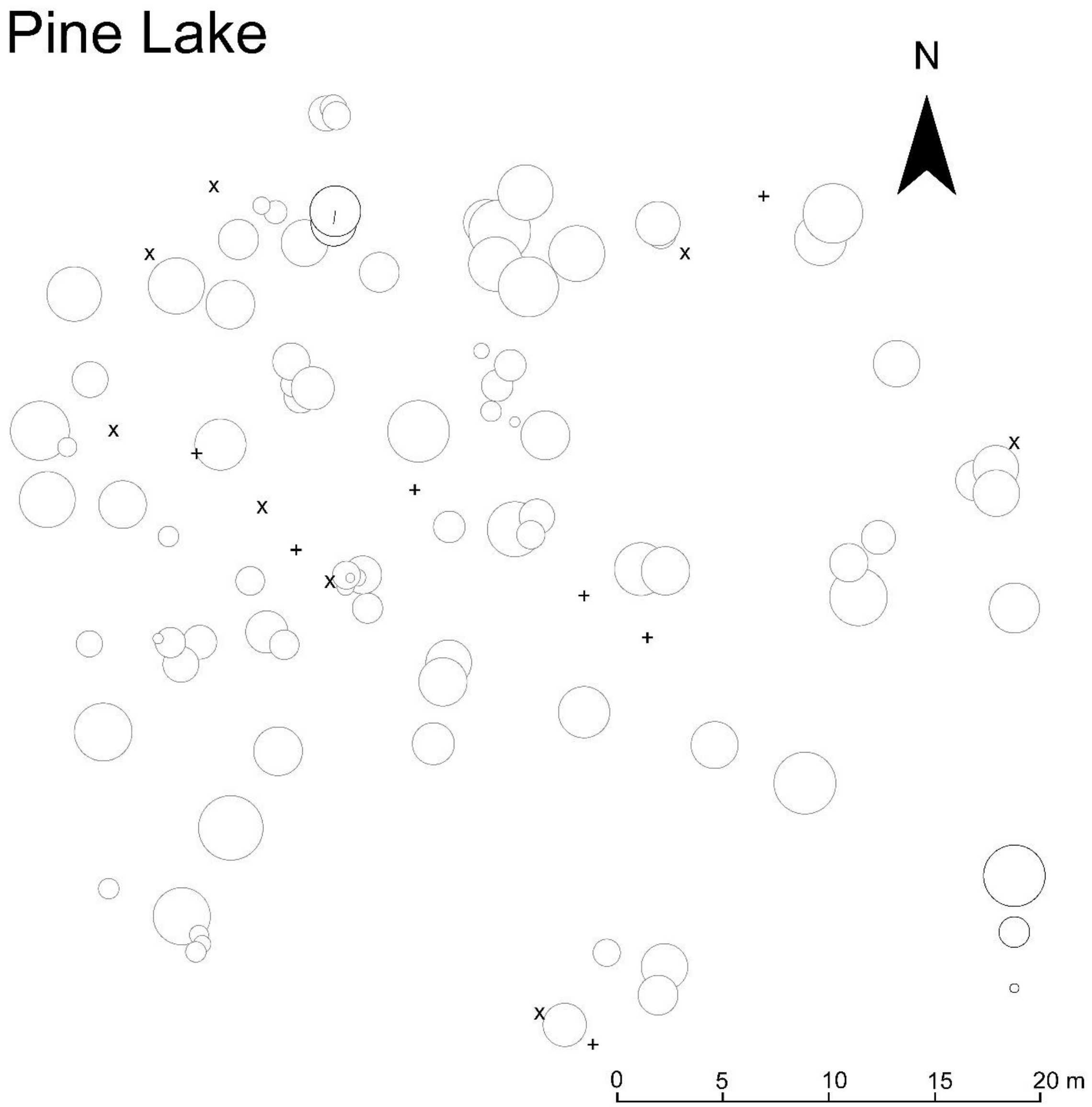

Supplementary Figure S5. The spatial distribution of the ramets of each identified multi-locus lineages (MLLs) and singletons in the Pine Lake plot. See caption of Figure S2 for explanation of figure. Eight dead stems are indicated by ‘x’ symbols and seven individuals with missing data are shown by crosses. The size of the shapes is proportional to the stem diameter with the diameter being a maximum 124.5 cm in this plot to a minimum of 2 cm.

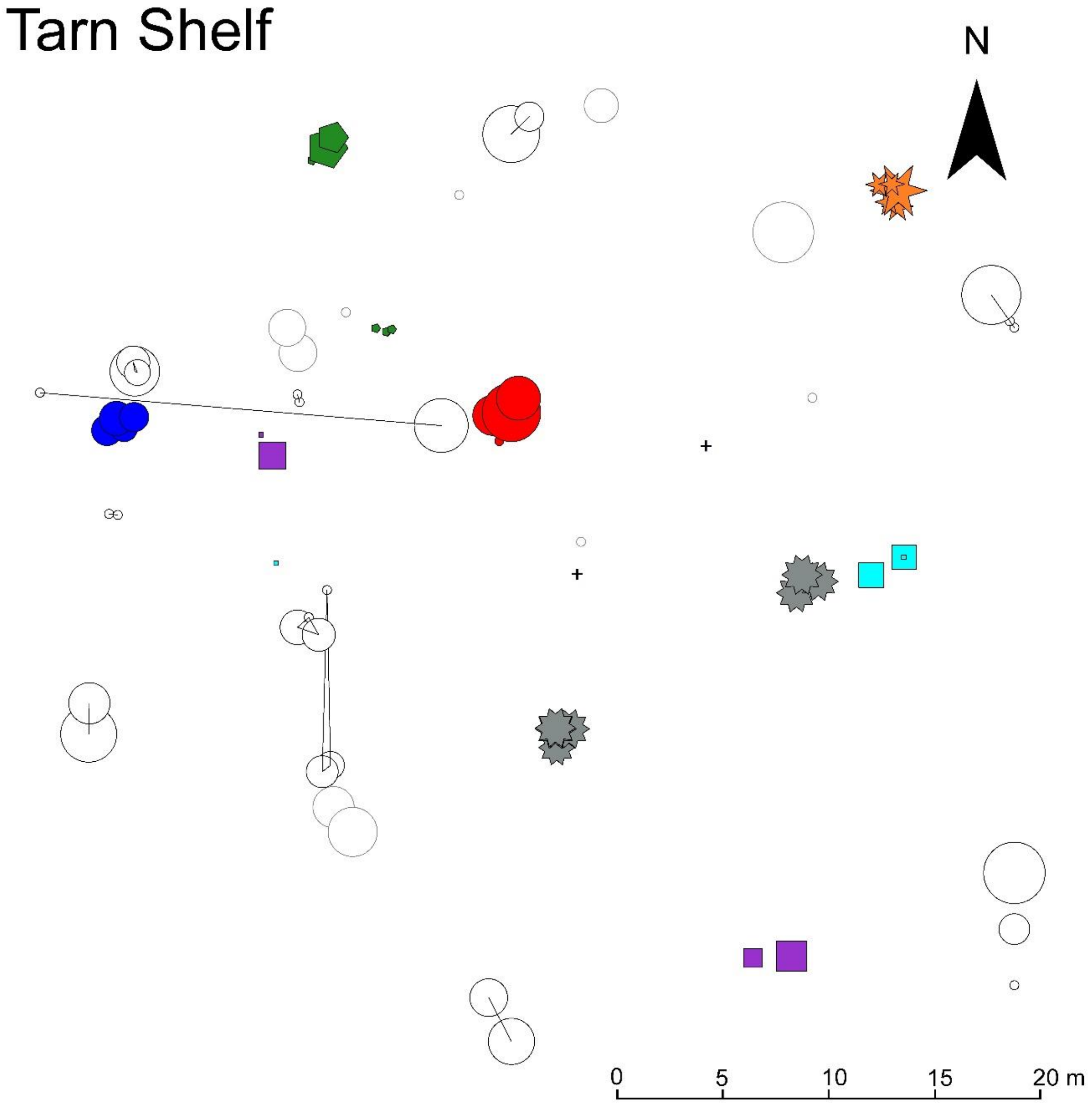

Supplementary Figure S5. The spatial distribution of the ramets of each identified multi-locus lineages (MLLs) and singletons in the Tarn Shelf plot. See caption of Figure S2 for explanation of figure. Two individuals with missing data are shown by crosses. No dead individuals were present. The size of the shapes is proportional to the stem diameter with the diameter being a maximum 95 cm in this plot to a minimum of 2 cm.

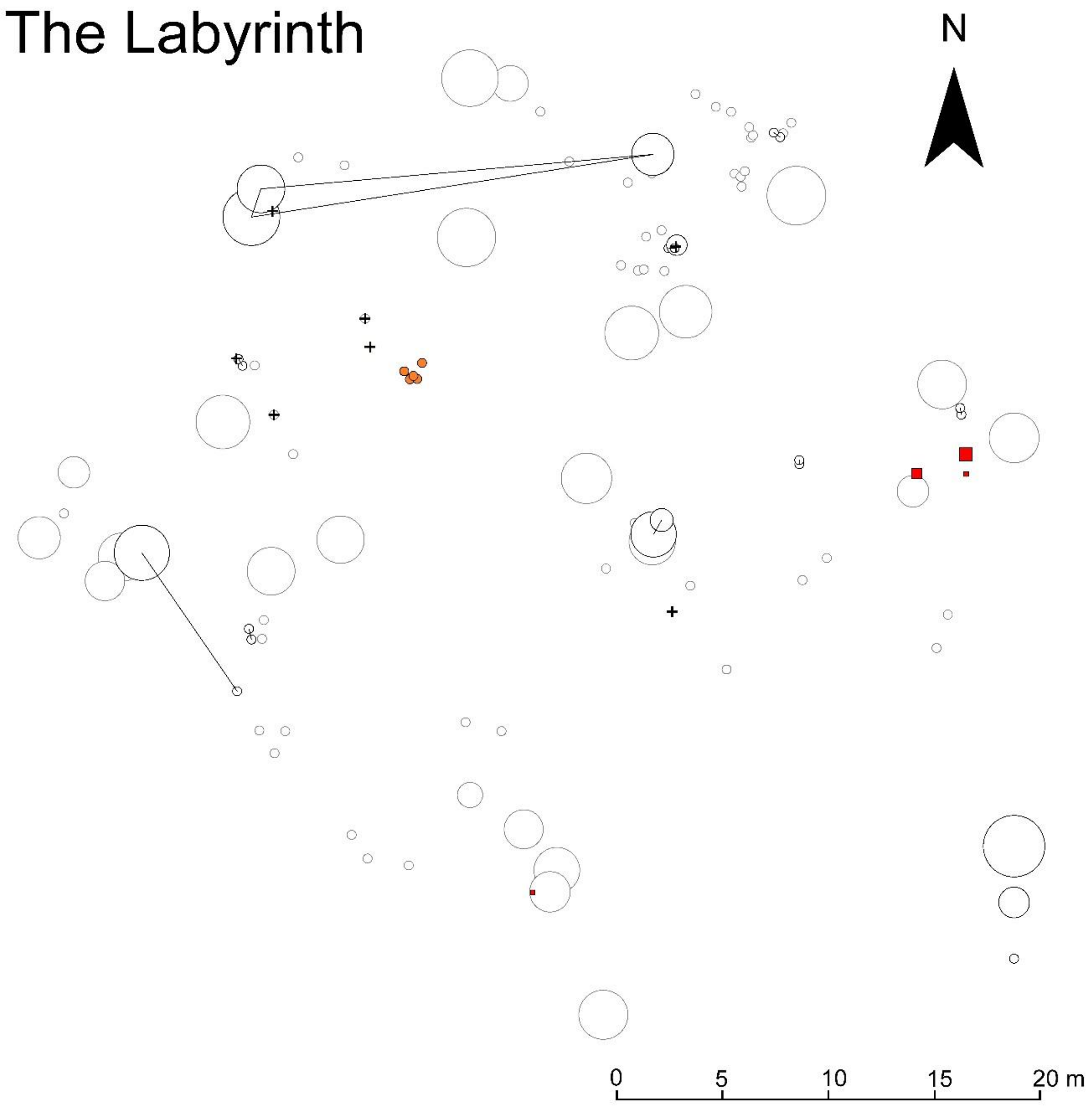

Supplementary Figure S6. The spatial distribution of the ramets of each identified multi-locus lineages (MLLs) and singletons in The Labyrinth plot. See caption of Figure S2 for explanation of figure. Eight individuals with missing data are shown by crosses. No dead individuals were present. The size of the shapes is proportional to the stem diameter with the diameter being a maximum 80.6 cm in this plot to a minimum of 2 cm.
